# Place-Based Diminished Returns of Economic Resources in Rural America: A Framework for Understanding Geography-Conditioned Inequality

**DOI:** 10.31586/jsmhes.2025.6067

**Published:** 2025-10-01

**Authors:** Shervin Assari, John Ashley Pallera, Babak Najand, Mojgan Azadi, Hossein Zare

**Affiliations:** 1 Department of Psychiatry, Charles R. Drew University of Medicine and Science, Los Angeles, CA, United States; 2 Department of Internal Medicine, Charles R. Drew University of Medicine and Science, Los Angeles, CA, United States; 3 Department of Family Medicine, Charles R. Drew University of Medicine and Science, Los Angeles, CA, United States; 4 Department of Urban Public Health, Charles R. Drew University of Medicine and Science, Los Angeles, CA, United States; 5 Marginalization-Related Diminished Returns (MDRs) Center, Los Angeles, CA, United States; 6 Charles R. Drew University of Medicine and Science, Los Angeles, CA, United States; 7 Montgomery College, Montgomery County, Rockville, MD, USA.; 8 Department of Health Policy and Management, Johns Hopkins Bloomberg School of Public Health, Baltimore, MD, United States; 9 School of Business, University of Maryland Global Campus (UMGC), Adelphi, United States

**Keywords:** Rural Health Disparities, Socioeconomic Status, Diminished Returns, MDRs, Place-Based Inequality, Geographic Context, Structural Inequality, Education, Parental Education, Health Equity, Social Determinants of Health, Rural America, Spatial Disadvantage, Contextual Moderators

## Abstract

**Background:**

Socioeconomic status (SES) is widely associated with improved health, behavioral, and educational outcomes. However, emerging research suggests that these benefits are not uniformly experienced across populations or contexts. The theory of Marginalization-related Diminished Returns (MDRs) has primarily focused on racial and ethnic disparities, showing that individuals from racially marginalized groups often experience weaker protective effects of SES. There is a lack of evidence on geography—particularly rural residence—as a moderator of SES effects.

**Objective:**

This review explores how place, especially rural contexts in the U.S., shapes the extent to which SES translates into improved outcomes. We extend the MDRs framework to include place-based and geography-based marginalization, arguing that even among non-Hispanic White populations, rural residence can lead to diminished returns on education, income, and other forms of capital.

**Content:**

Drawing on theoretical models such as Fundamental Cause Theory and Bronfenbrenner’s Ecological Systems Theory, and synthesizing empirical findings from studies of academic achievement, substance use, and educational aspirations, this review highlights how structural disadvantages in rural areas weaken the effectiveness of individual and family-level resources.

**Conclusion:**

Rural health and educational disparities are not solely due to a lack of resources but may also reflect systemic conditions that erode the value of existing resources. Policy interventions must be place-aware and address the contextual constraints that limit opportunity. Future research should more explicitly test how geography moderates the effects of SES across a range of outcomes and populations.

## 1. Background

Health disparities between rural and urban populations have been widely documented for decades both in the United States [1,2] and Worldwide [3,4]. Individuals living in rural areas experience higher rates of chronic disease, mental health challenges, substance use, disability, and premature mortality [5]compared to their urban counterparts. These disparities are often attributed to a combination of factors, including geographic isolation, reduced access to healthcare providers, hospital closures[6], higher levels of poverty, and fewer educational and employment opportunities. Despite a growing body of research and policy initiatives aimed at closing rural-urban gaps, many of these disparities remain persistent or have even widened in recent years. However, most rural health research has focused on lack of resources in rural communities [1,7], such as fewer clinics or lower funding, rather than examining how existing resources may also function differently across geographic contexts. This paper aims to shift the conversation from rural scarcity to rural inequity by exploring how place itself can alter the impact of individual and family-level socioeconomic resources—ultimately introducing the concept of *place-based diminished returns*.

## 2. Paper structure

As shown by Table 1, this paper follows an evidence-informed narrative that is organized into six major sections. The first section, *Foundational Concepts* (Sections 1–5), introduces the protective role of socioeconomic status (SES), the significance of place as a social determinant of health, and how geographic context shapes access to opportunity, with special attention to the structural and demographic characteristics of rural communities and rural states. The second section, *Intersecting Marginalities* (Sections 6–10), explores how race and place intersect to shape health and educational outcomes. It draws upon theories such as Fundamental Cause Theory and Bronfenbrenner’s Ecological Systems Model to argue that rural residence should be considered an independent axis of inequality. The third section, *Mechanisms of Rural Disadvantage* (Sections 11–15), examines how structural limitations—such as reduced access to services, under-resourced institutions, limited labor markets, and low-quality education—contribute to the erosion of SES effectiveness in rural contexts.

The fourth section, *Theory of MDRs and Its Application* (Sections 16–20), introduces the Marginalization-related Diminished Returns (MDRs) framework, which has traditionally been applied to race and ethnicity, and extends it to geography. This section also contrasts the concept of place-based MDRs with traditional interpretations of rural health disparities, highlighting the importance of considering contextual moderators. The fifth section, *Empirical Evidence of Rural MDRs* (Sections 21–25), presents data from national studies such as Add Health and Monitoring the Future (MTF), illustrating that even among non-Hispanic White youth, parental education confers weaker protective effects in rural or disadvantaged places. Finally, the sixth section, *Implications and Forward Thinking* (Sections 26–30), outlines the policy relevance of these findings, calls for future research that better integrates place into disparity frameworks, discusses limitations of the existing evidence base, and concludes with a call for equity-centered, place-aware interventions to address compounded disadvantage in rural America.

### 2.1. Socioeconomic Status as a Determinant of Health and Well-being

Socioeconomic status (SES)—often measured through education, income, and occupation—is a key predictor of health, development, and life opportunities[8]. Across various studies, higher SES is typically associated with better health, academic performance, and lower risk behaviors. Education contributes to greater knowledge of health-promoting behaviors, higher health literacy, and better employment prospects, while income affords access to healthcare, safe environments, and material resources that buffer stress[9–12]. However, the benefits of SES are not always equally distributed across all populations or settings. The traditional assumption that more resources yield better outcomes may not hold under certain structural or contextual conditions, including geographic location.

### 2.2. The Role of Place as a Social Determinant of Health

Place—or where one lives—has been increasingly recognized as a crucial social determinant of health[13]. Geography can influence access to healthcare, educational quality, job markets, public transportation, and exposure to environmental risks[14–16]. Structural features such as residential segregation, economic disinvestment, and policy neglect can shape entire communities’ life chances. Thus, place is not just a backdrop but an active force in shaping the opportunities and constraints that individuals face. The same individual characteristics or resources can yield vastly different outcomes depending on the place in which one resides.

### 2.3. How Place Shapes Health Outcomes

Health outcomes are deeply embedded in the broader geographic, social, and economic environment [47–50]. Rural areas, for example, often lack essential healthcare services, mental health providers, and emergency care facilities [51]. These areas may also have limited infrastructure, healthy food, and fewer opportunities for physical activity, all of which impact health [52]. Educational institutions in rural communities may suffer from underfunding, teacher shortages, and low college preparatory resources [53]. Together, these contextual disadvantages can erode the potential benefits of individual or household resources and contribute to widening disparities in health and achievement.

### 2.4. Demographics of Rural Areas in the United States

The U.S. Census Bureau classifies areas as either urban or rural based on specific geographic and population characteristics [54]. Urban areas are defined by densely built environments that include housing, businesses, and other types of development [55]. These areas are identified following each decennial census using established criteria applied to census data. In contrast, rural areas include all regions that fall outside the boundaries of urban classifications [56]. For the 2020 Census, urban areas were defined as having a densely populated core of census blocks that meet certain thresholds for population or housing unit density. Surrounding areas with related non-residential development are also included. To be designated as urban, a location must contain at least 2,000 housing units or a population of 5,000 or more [57].

Rural communities in the U.S. are often perceived as racially and ethnically homogenous, yet they are increasingly diverse [17]. While non-Latino White populations still make up the majority in many rural areas, there are growing numbers of Latino, Black, Native American, and immigrant residents. Rural America also tends to skew older and experiences higher rates of disability [58]. Economic structures in these regions are often built around agriculture, manufacturing, or resource extraction—industries that have faced economic downturns. These demographic and structural features shape the kinds of resources available, the level of need, and the potential effectiveness of health and social interventions [59–61].

### 2.5. The Landscape of Rural States and Policy Environments

**Figure 1.**
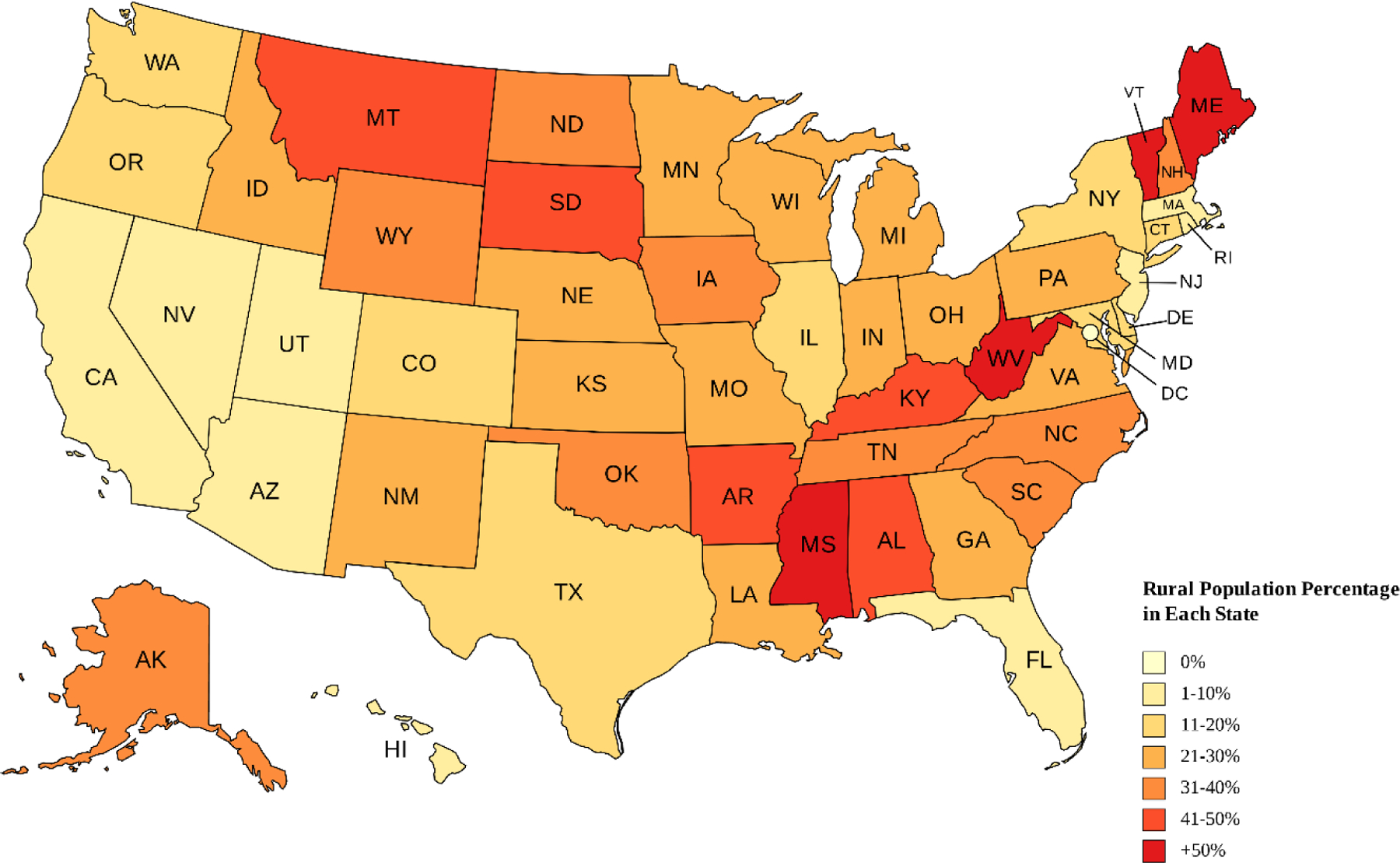
Rural Populations in the USA [62]

Figure 1 shows the distribution of rural populations in the USA. Rural states in USA are listed in Table 2. States with large rural populations, such as West Virginia, Mississippi, Kentucky, and parts of the Midwest and South, often face economic and health-related challenges. These states may have higher rates of uninsured residents, lower Medicaid expansion uptake, and fewer public investments in education and infrastructure [63,64]. Policy environments in rural states often reflect broader political ideologies that may deprioritize safety net programs and progressive taxation, further limiting the public sector’s capacity to buffer structural inequalities [65]. These factors make rural states a key context in which to study diminished returns of SES and to test place-based theories of inequality.

### 2.6. SES and Rural Residence: Intersecting Marginalizations

SES, race/ethnicity and geography are intertwined in ways that complicate our understanding of disadvantage. While rural America is often portrayed as predominantly low SES White, many rural regions are home to racially and ethnically minoritized populations, including Black communities in the Deep South, Native populations in the Plains and Southwest, and Latino and immigrant populations in agricultural hubs [66]. These populations may face both race-based and place-based marginalization, leading to compounded disadvantage (low-resource neighborhoods or communities)[67]. Even non-Hispanic White individuals living in rural areas may encounter economic and social exclusion due to regional underinvestment [68]. The convergence of race and place challenges simplistic narratives and calls for intersectional analyses of health inequality [69].

### 2.7. Place as a Fundamental Cause

The Fundamental Cause Theory [18–21] posits that SES functions as a root cause of health disparities because it provides flexible resources—money, knowledge, power, and social connections—that can be used to avoid risks and adopt protective strategies. However, the theory also acknowledges that the effectiveness of these resources may vary based on structural conditions. In rural areas, even individuals with higher SES may find fewer health-promoting opportunities to leverage their resources. The very mechanisms by which SES exerts its protective effects—access to providers, education, or employment—may be weaker, unavailable, or geographically distant in rural settings, contributing to persistent disparities.

### 2.8. Place as a Social Determinant of Health

In rural areas, several social determinants of health—such as education, employment, housing, transportation, and healthcare access—are structurally compromised[22]. Health care deserts, food insecurity, inadequate public infrastructure, and weak social safety nets create a challenging context that can erode individual and family resilience [70]. Even when families have higher SES, these contextual constraints may neutralize or reduce the advantages typically associated with education and income. For example, a highly educated family may still live in a town with no hospital, limited broadband, or failing schools, limiting the translation of SES into improved outcomes.

### 2.9. Bronfenbrenner’s Ecological Systems Theory and Place-Based Inequality

Bronfenbrenner’s Ecological Systems Theory [23–26] offers a useful framework for understanding how place-based disadvantage affects individuals across nested levels of influence. At the microsystem level, family SES and parental involvement shape youth outcomes. At the meso-system and macro-system levels, community resources, institutional structures, and sociopolitical environments exert broader influences. Rural areas may feature compromised mesosystemic interactions—such as weak links between schools and community services—that constrain positive developmental trajectories. This ecological model suggests that without supportive settings at multiple levels, the benefits of individual and family-level SES may not fully manifest.

### 2.10. Rural Residence as a Source of Health Inequities

Living in a rural area is independently associated with a host of poor health outcomes, including higher rates of chronic disease, mental health disorders, disability, and premature death [71]. These outcomes are often attributed to structural barriers such as physician shortages, long travel distances to care, under-resourced hospitals, and limited public health infrastructure. In addition to material constraints, cultural and political dynamics in some rural communities may discourage help-seeking or stigmatize mental health care. When such barriers are present, even higher income or education may not translate into better health, suggesting that rural residence itself functions as a site of diminished return on economic resources.

### 2.11. Mechanisms That Shape Rural Health Disparities

A wide array of interrelated mechanisms contributes to health disparities in rural settings, even when SES levels are comparable to those in urban areas. Rural populations often face lower overall SES [72], but even when SES is high, individuals may still experience lower access to healthcare, educational opportunities, and employment [39,40]. Challenges such as higher rates of poverty, food insecurity, and unemployment intersect with infrastructural limitations like inadequate transportation, school closures, and broadband gaps. In these contexts, individual resources may not translate into meaningful differences in exposure or opportunity, thereby reducing the health returns typically associated with education and income [39,40].

### 2.12. Reduced Access to Services and Information

One of the central mechanisms behind diminished SES returns in rural areas is reduced access—not just to healthcare, but to a broad range of social services, educational resources, and even timely information[73]. Many rural residents live far from health centers, pharmacies, or mental health providers, and even internet access may be limited, restricting access to telehealth or remote education. These structural limitations disproportionately affect families regardless of SES, but particularly undermine the ability of high-SES families to leverage their resources [74]. The spatial isolation common to rural living dilutes the buffering effect that SES often provides in more resource-rich settings.

### 2.13. Employment and Economic Opportunity Constraints

Rural labor markets are often characterized by limited employment opportunities, lower wages, and unstable work [75]. Even individuals with higher education levels may face difficulty finding jobs that match their qualifications, leading to underemployment or long commutes. This mismatch between educational attainment and labor market opportunities can lead to economic strain, dissatisfaction, and stress, all of which can impact health and well-being. The absence of high-quality employment options reduces the motivational returns on education and blunts the intergenerational benefits often associated with upward mobility, especially in isolated or economically stagnant rural regions.

### 2.14. Educational Resource Gaps and School Quality

In rural areas, schools frequently face challenges including lower funding, difficulty attracting and retaining qualified teachers, and fewer advanced coursework or extracurricular opportunities. As a result, even children from highly educated families may not benefit from the enriched learning environments typically found in urban or suburban schools [76]. Parents may have the aspiration and knowledge to support their children’s educational advancement, but systemic constraints—such as lack of access to college preparatory resources [77] or counseling—may obstruct the translation of those advantages into academic outcomes. Thus, school quality serves as another critical site of diminished returns for families in rural communities.

### 2.15. When the Same SES Resources Yield Different Outcomes Across Places

The concept of Minorities’ Diminished Returns suggests that identical levels of SES resources do not yield identical outcomes across all contexts [38–40]. In rural settings, this pattern becomes apparent when high parental education or household income does not lead to better health, behavioral, or academic outcomes. For example, two children may have college-educated parents, but the child in a rural town may attend a poorly resourced school, live in a healthcare desert, or lack exposure to diverse role models or career pathways. Place, therefore, becomes a moderator that shapes the translation of resources into outcomes, reinforcing spatial inequalities even within SES-matched populations.

### 2.16. Introduction to Minorities’ Diminished Returns (MDRs)

The theory of Minorities’ Diminished Returns (MDRs) [27] suggests that socioeconomic resources—such as education and income—yield weaker health, behavioral, and economic outcomes for marginalized populations than for more advantaged groups. First developed to explain racial and ethnic disparities, MDRs offer a framework for understanding why simply equalizing access to resources may not eliminate inequality. The core idea is that systemic barriers, and contextual inequalities may reduce the efficacy of those resources for marginalized groups. This concept has been applied to explain disparities in areas such as mental health, academic achievement, substance use, and economic stability.

### 2.17. MDRs Most Commonly Documented for Race and Ethnicity

Most existing MDRs research has centered on race and ethnicity, showing that Black, Latino, and Asian individuals tend to receive less benefit from education, income, and employment than their White counterparts[27]. For example, while higher parental education is associated with better school performance for White youth, this effect is often smaller for youth of color. These patterns persist across a range of health and behavioral outcomes and are not fully explained by differences in absolute SES levels. Instead, they reflect systemic barriers, such as racism, residential segregation, and institutional bias, that diminish the returns on resources for racialized groups.

### 2.18. MDRs Extend Beyond Race to Other Marginalized Groups

While the original MDRs literature focused on race and ethnicity [27], the framework has expanded to other forms of marginalization including rural residence [39,40]. Studies have shown diminished returns for LGBTQ+ individuals[28,29], immigrants [30–36], and other marginalized groups [37]. For instance, sexual minority youth may not experience the same protective effects of parental education or family income on mental health outcomes [29]. Immigrants with high educational attainment may still face labor market exclusion and wage penalties [30–36]. These extensions suggest that MDRs are not unique to race but rather a broader pattern linked to structural marginalization, discrimination, and social exclusion.

### 2.19. Place-Based MDRs: A New Frontier in the Study of Health Inequities

Place-based MDRs suggest that geography—particularly rural residence—can function similarly to other axes of marginalization. Just as race or sexuality can reduce the protective effects of SES, so too can living in a disadvantaged place[38–40]. Emerging studies indicate that non-Latino White youth in rural or low-resource neighborhoods may not benefit as much from parental education as their urban counterparts. This expands the MDRs framework beyond identity-based marginalization to include spatial inequities, offering a new lens for understanding rural disadvantage not just as a lack of resources, but as a site of weakened resource efficacy.

### 2.20. Place-Based MDRs Differ from Traditional Rural Health Disparity Narratives

Some rural health disparity research tends to emphasize a lack of resources: fewer doctors, lower incomes, poor infrastructure[63,74]. Place-based MDRs, in contrast, suggest that even when resources are present—such as well-educated families or decent incomes—the benefits of those resources may not fully translate into improved outcomes due to contextual constraints. This is a shift from seeing rural disadvantage as a problem of scarcity to recognizing it as a problem of blocked opportunity. This perspective highlights structural barriers within rural environments that dilute the power of SES, rather than simply noting its absence.

### 2.21. Why the Effects of SES May Be Reduced in Rural Areas

Several factors help explain why the protective effects of SES are weaker in rural settings. First, even when families have economic resources or high educational attainment, they may still face structural and environmental barriers that limit their options. For example, children of highly educated parents in rural areas may still struggle to access high-quality schools or a highly educated patient in rural areas may face challenges to receive timely medical care [68]. Second, social capital and professional networks tend to be more limited in rural areas, reducing the opportunities for mentorship, internships, or academic advancement. Third, rural stigma, underinvestment, and geographic isolation all contribute to an environment in which SES resources have reduced utility [76–80].

#### Other Structural Barriers in Rural Areas

Beyond education and occupation, rural environments are often shaped by structural limitations that may also influence health, well-being, and cognitive outcomes. Limited access to high-speed internet, for instance, can constrain educational opportunities, social connection, and access to telehealth or digital resources[73]. Similarly, a lack of libraries or bookstores reduces exposure to books and other learning materials, limiting both lifelong learning and early literacy development.

Healthy food access is another challenge, as many rural areas qualify as “food deserts” where residents face difficulties finding affordable and nutritious options. This scarcity of fresh produce and reliance on processed foods may increase risks for chronic conditions such as obesity, diabetes, and cardiovascular disease. In addition, access to safe and affordable recreational facilities is often restricted [78]. The absence of gyms, fitness centers, or even walkable community spaces reduces opportunities for physical activity, further contributing to health disparities [79].

Taken together, these structural barriers illustrate rural context may create an environment where individuals’ employment, education or income may face limitations in translation into better health and economic outcomes [38–40]. These factors are consistent with the framework of diminished returns, showing that the environment surrounding individuals can restrict the benefits usually associated with socioeconomic resources.

### 2.22. MDRs in Rural Areas: Empirical Support from Educational Outcomes

Recent studies have begun documenting place-based MDRs by analyzing how the effects of SES on educational outcomes vary by geography[38]. One such study using the Add Health dataset found that among non-Hispanic White adolescents, the association between parental education and school performance was significantly weaker in lower-quality neighborhoods. Even when controlling for race and income, neighborhood disadvantage moderated the impact of parental education. This finding suggests that contextual factors like segregation, concentrated poverty, and environmental disorder diminish the protective effects of family-level SES, even among groups not traditionally viewed as disadvantaged.

### 2.23. Case Example #1: Academic Aspirations Among Rural Adolescents

Data from the Monitoring the Future (MTF) study of 12th-grade students further illustrates rural MDRs[39]. The study found that while parental education was generally associated with greater aspirations for graduate or professional education, this effect was weaker in rural areas than in urban or suburban settings. In rural contexts, even high parental education did not consistently translate into higher educational ambition[39]. This may be due to limited role models, weak school-to-college pipelines, or lower perceived returns on advanced education in rural labor markets. The findings support the idea that place moderates how far individual or familial resources can carry a young person’s aspirations.

### 2.24. Case Example #2: Parental Education and Nicotine Use Among Rural Youth

Another example comes from a recent analysis of the 2024 Monitoring the Future data, which found that parental educational attainment was less protective against youth use of oral nicotine products—such as pouches, gummies, and candies—in marginalized environments[40]. Even among non-Latino White 12th graders, the benefits of higher parental education were attenuated in contexts of neighborhood disadvantage and high school-level poverty. These findings align with the MDRs framework and suggest that rural and economically disadvantaged places can disrupt the typical relationship between parental education and lower substance use risk, even when race and ethnicity are held constant.

### 2.25. Interpreting the Evidence: Contextual Moderation of Parental Education Effects

Across these examples, a consistent theme emerges: the environment in which individuals live conditions how effectively their SES resources protect against risk or promote well-being. In rural areas marked by economic disinvestment and limited institutional support [80], high parental education does not offer the same level of protection as it does in more resource-rich settings. These findings do not suggest that parental education is unimportant; rather, they point to the need for place-sensitive analyses and policies that recognize how geography can shape, and sometimes diminish, the benefits of individual and family-level resources.

### 2.26. Policy Implications: Beyond Resource Distribution

The evidence of place-based diminished returns calls for a policy shift that goes beyond equalizing access to socioeconomic resources. While efforts to increase educational attainment and reduce poverty remain important, they may be insufficient on their own if rural contexts continue to suppress the utility of those gains. Policy strategies must also address the structural barriers embedded in rural communities—such as underfunded schools, lack of public transportation, healthcare shortages, and limited economic opportunity. Investments must be made in the institutions and infrastructures that allow SES to “work” more effectively in rural settings. This may include broadband expansion, college pipeline programs, and rural health incentives that support systemic capacity.

Place-based interventions, policies, and programs are essential for addressing the unique structural and contextual factors that shape health and social outcomes within specific geographic settings [41–43]. Unlike broad, one-size-fits-all strategies, place-based approaches acknowledge that the effectiveness of resources and interventions can vary dramatically depending on local conditions such as infrastructure, access to services, economic opportunities, and social capital. In rural or under-resourced communities, for example, high educational attainment or income may not lead to the same benefits observed in more suburban, resource-rich settings due to structural and systemic barriers like healthcare shortages, poor school quality, or limited job markets. Tailoring interventions to the specific needs and challenges of a place can enhance their impact, promote equity, and ensure that investments in education, health, and economic development translate into meaningful improvements for residents. By accounting for the role of geography in shaping opportunity and outcomes, place-based strategies offer a more just and effective pathway for reducing disparities and fostering community resilience.

Place can serve as a powerful lens for tailoring interventions, much like the way interventions are often adapted based on gender, race, ethnicity, or education level. Recognizing that the effectiveness of a program or policy may vary by geographic context allows for more precise and equitable responses [44,45]. Tailoring interventions to place means accounting for the unique social, economic, and infrastructural realities that shape residents’ access to resources and their ability to benefit from them. In rural areas, for instance, the same health or educational intervention used in an urban setting may require significant adaptation to address barriers such as limited service availability, lower population density, or weaker institutional capacity.

Specific place-based adaptations—such as expanding distance learning opportunities, improving access to telehealth services, or enhancing transportation infrastructure—can help mitigate access challenges faced by populations in rural or underserved areas. These interventions are particularly valuable in regions where geographic distance, provider shortages, or inadequate public transit limit access to healthcare, education, and social services. By addressing structural barriers tied to location, such approaches can help ensure that individuals in disadvantaged or isolated settings are not left behind. Ultimately, incorporating place as a factor in designing and implementing interventions supports more inclusive policies that respond directly to the lived realities of diverse communities.

### 2.27. Future Research Directions: Centering Place in MDRs Studies

Future research should more intentionally incorporate geographic context into the study of diminished returns. While much of the existing MDRs literature has focused on racial and ethnic disparities, rurality represents an understudied but crucial moderator of socioeconomic effects. Researchers should consider including interaction terms between SES and place in statistical models, stratifying analyses by rural-urban status, and using multi-level designs that can isolate individual and contextual influences. There is also a need for longitudinal studies that examine how diminished returns unfold across time, particularly in developmental and intergenerational contexts. Incorporating qualitative insights from rural communities themselves may also shed light on mechanisms and resilience strategies.

### 2.28. Limitations of Existing Evidence and This Review

Despite growing interest in place-based MDRs, the current body of evidence remains limited in scope and depth. Most findings to date rely on secondary analysis of large datasets, which may not be designed to capture the full complexity of rural contexts or the nuances of place-based disadvantage. Additionally, measures of rurality, neighborhood quality, and access often vary across studies, making comparisons difficult. This review is also limited by the focus on U.S. rural populations, with less attention to how place-based MDRs may operate in global settings. More research is needed to confirm findings, test mechanisms, and guide tailored interventions.

### 2.29. MDRs and a Paradigm Shift to Study Rural Health Disparities

Understanding rural disparities through the MDRs framework [46]allows for a reframing of the problem. Rather than viewing rural communities solely as lacking resources, we begin to see them as places where systems have less success to amplify or convert those resources into outcomes. This subtle but powerful shift in thinking moves us from deficit-focused models toward structural analyses that highlight opportunity blockages. It also helps explain why even well-resourced rural families may struggle to achieve the same health or educational outcomes as similarly resourced families elsewhere. The MDRs framework thus adds explanatory depth to our understanding of rural inequality.

### 2.30. Limitations

While this paper advances the understanding of place and health, as well as their policy implications, several limitations should be acknowledged. First, this is not a systematic review, and therefore the process did not follow structured protocols such as PRISMA guidelines. Consequently, some relevant studies may have been missed, particularly those published in non-indexed journals or in disciplines beyond public health and social epidemiology. Second, the literature reviewed was selective and intended to highlight major patterns across domains rather than provide exhaustive coverage. Third, much of the cited evidence comes from U.S.-based studies, which may limit the generalizability of conclusions to other national or cultural contexts, even though international research increasingly demonstrates similar patterns. Finally, the synthesis relied on published studies, which may be affected by publication bias toward reporting disparities, while null or contrary findings may be underrepresented. Despite these limitations, this paper offers a broad, evidence-informed synthesis that underscores the literature on how rural residence and place-based factors can reduce the health returns of education and other economic resources. It also emphasizes the urgency of implementing policy solutions aimed at dismantling place-based health inequities.

### 2.30. Conclusion: The Need for Place-Aware Equity Interventions

In conclusion, place matters—not only as a determinant of health and opportunity but also as a context that shapes the returns and utility of socioeconomic resources. Rural areas, while diverse and resilient, are often structured in ways that suppress the returns on education, income, and employment. Place-based diminished returns offer a compelling extension of the MDRs theory, one that broadens our understanding of inequality beyond identity to include geography. Addressing these disparities will require coordinated efforts in research, policy, and practice to ensure that families in rural communities not only have access to resources but also the opportunity to benefit from them. Only then can we move toward a more equitable society that recognizes and corrects the compounded disadvantages of both place and identity.

## Figures and Tables

**Table 1.** Areas Covered by the Paper

Section	Sections	Description
1. Foundational Concepts	1–5	Introduces the protective role of SES, the importance of place as a social determinant of health, and how place shapes access to opportunity, with a focus on the unique characteristics of rural communities and rural states.
2. Intersecting Marginalities	6–10	Discusses how SES and place interact, draws on Fundamental Cause Theory and Bronfenbrenner’s ecological model, and identifies rural residence as an independent source of health inequity.
3. Mechanisms of Rural Disadvantage	11–15	Explores how low access, weaker institutions, poor labor markets, and underfunded education systems act as mechanisms that reduce the effectiveness of SES in rural areas.
4. Theory of MDRs and Its Application	16–20	Introduces the MDRs framework, traditionally used in racial/ethnic disparities research, and expands it to geographic place. Discusses how place-based MDRs differ from traditional rural health narratives.
5. Empirical Evidence of Rural MDRs	21–25	Reviews real-world examples from national datasets (Add Health, MTF), showing that even among non-Hispanic White youth, parental education offers weaker protection in rural or disadvantaged places.
6. Implications and Forward Thinking	26–30	Outlines implications for policy, identifies future research needs, discusses limitations, and concludes with a call for equity interventions that account for geography as a structuring force in inequality.

**Table 2.** US States with highest % of Population Living in Rural Areas

State	% of Population Living in Rural Areas
Maine	61.30%
Vermont	61.10%
West Virginia	51.30%
Mississippi	50.70%
Montana	44.10%
Arkansas	43.80%
South Dakota	43.40%
Kentucky	41.60%
Alabama	41.00%
North Dakota	40.10%
